# Impact of COVID-19 on Renal Function: A Multivariate Analysis of Biochemical and Immunological Markers in Patients

**DOI:** 10.7759/cureus.22076

**Published:** 2022-02-10

**Authors:** R Panimathi, Ezhil Gurusamy, S Mahalakshmi, K Ramadevi, G Kaarthikeyan, Sukumaran Anil

**Affiliations:** 1 Institute of Biochemistry, Madras Medical College and Research Institute, Chennai, IND; 2 Saveetha Dental College, Saveetha Institute of Medical and Technical Sciences (SIMATS), Chennai, IND; 3 Department of Dentistry, Oral Health Institute, Hamad Medical Corporation, Doha, QAT; 4 College of Dental Medicine, Qatar University, Doha, QAT

**Keywords:** sars-cov-2, renal function tests, serum biomarkers, laboratory diagnosis, covid-19, coronavirus disease

## Abstract

Introduction

There have been tremendous continuous efforts to understand the broad spectrum of disease and its sequelae since the start of the coronavirus disease 2019 (COVID-19) pandemic. Several studies have identified biomarkers that correlate with multiple organ failure in COVID-19 patients. The purpose of our study was to evaluate COVID-19-associated kidney injury.

Methods

This retrospective cross-sectional study was conducted at the Institute of Biochemistry, Madras Medical College, by reviewing the electronic records of 1,000 reverse transcription-polymerase chain reaction (RT-PCR)-confirmed COVID-19-positive patients admitted at the COVID-19 care center. Data were extracted from the case records of 1,000 RT-PCR-positive patients with different CT chest grades plus comorbid conditions such as type 2 diabetes mellitus (T2DM), systemic hypertension (SHT), coronary artery disease (CAD), chronic obstructive pulmonary disease (COPD), and cerebrovascular accident (CVA) as Group I (n = 500). Group II (n = 500) comprised of COVID-19-positive patients with no comorbid conditions. The data were recorded from all the patients at the time of admission, prior to starting treatment. Patients with comorbid and non-comorbid conditions were compared according to different CT grades.

Results

COVID-19 patients with different CT grades showed a significant relationship with creatinine, sodium, potassium, C-reactive protein (CRP), ferritin, total protein, and albumin with p-values of 0.04, 0.01, 0.02, 0.000, 0.00, 0.00, and 0.000, respectively, in Group I. In Group II, with various grades of CT changes, the neutrophil-lymphocyte ratio (NLR) and creatinine showed no significance. The sodium, potassium, CRP, ferritin, total protein, and albumin showed low significance with the chest CT grades.

Conclusions

Our study demonstrated that COVID-19 can cause mild to moderate renal impairment in COVID-19 patients. Multiple factors contributed to this, such as the higher angiotensin-converting enzyme 2 (ACE2) expression on kidney cells, microinflammation, increased blood clotting, and probable direct infection of the kidney. A high NLR, increased inflammatory markers, and altered renal function analytes such as urea, creatinine, sodium, potassium, total protein, and albumin also confirmed this.

## Introduction

The acute manifestations of coronavirus disease 2019 (COVID-19) have been well-documented and severe COVID-19 can cause multi-organ injury and failure [1-3]. The presence of hypertension (HT) and type 2 diabetes mellitus (T2DM) worsened COVID-19 outcomes in India with a high number of tuberculosis and pneumonia cases. Electrolyte imbalances were also one of the most common presentations of COVID-19 and arose as a complication during the course of illness [4,5]. The high prevalence of COVID-19 infection in HT patients could be attributed to the use of angiotensin-converting enzyme (ACE) inhibitors since this COVID-19 virus binds to angiotensin-converting enzyme 2 (ACE2) receptors to enter the target cells [6]. ACE inhibitors and angiotensin receptor blockers (ARBs) increase the expression of ACE2 receptors in liver cholangiocytes, kidney podocytes, and tubular cells, facilitating target organ infection and stimulating the progression of the disease in target organs, thereby leading to direct renal cell injury. It may also cause hypoperfusion and thrombotic microangiopathy by affecting endothelial cells. Inflammatory cytokines also induce acute kidney injury (AKI) and glomerulopathy [7].

When a diabetic patient develops COVID-19 infection, since the immune system is compromised, it is difficult for the patient to fight the viral infection and he or she likely experiences a prolonged recovery period. The virus may also thrive in an environment of elevated and fluctuating blood glucose levels and in the presence of T2DM complications [8]. COVID-19 infection has been associated with an altered antiviral host response, leading to rapid replication of the virus followed by a hyperinflammatory state, which causes dysregulation of the renin-angiotensin-aldosterone system (RAAS), precipitating acute lung injury and hypoxemia [9]. COVID-19 virus expresses proteins that inhibit interferon-1 production and delay the antiviral response by host cells. Subsequent delayed and persistent dysregulation of the interferon-1 response together with pro-inflammatory cytokines may cause excessive infiltration of monocytes/macrophages and neutrophils into the lung parenchyma.

These macrophages and polymorphonuclear neutrophils produce high levels of proinflammatory cytokines, such as interleukin 6 (IL-6), tumor necrosis factor (TNF), and other chemokines, promoting cytokine storms. Although the symptomatology ranges from fever, cough, shortness of breath, and other breathing difficulties, to nonspecific symptoms such as headache, fatigue, and muscle pain, X-ray and chest CT indicated lung pathologies that were related to the severity of infection [10]. Mild COVID-19 pneumonia presents with small subpleural, unilateral, or bilateral ground-glass opacities in the lower lobe that develop into a crazy-paving pattern and consequent consolidation. After two weeks, if not treated properly, the lesions become gradually absorbed, resulting in subpleural parenchymal bands [10].

Hyponatremia is the most common electrolyte disorder with a multifactorial etiology and is associated not only with pneumonia but also with gastrointestinal tract (GIT) symptoms of this infection. The most common cause of hyponatremia is the syndrome of inappropriate antidiuretic hormone secretion (SIADH). Elevated cytokines directly contribute to the impairment in osmoregulation, thereby precipitating hyponatremia. Hypokalemia described in a few cases might have resulted from hyperactivation of the RAAS, potassium loss, and anorexia, and secondarily from concurrent illness and tubular damage caused by ischemia. Alfano et al. [11] noted that the primary cause of hypokalemia was dependent on the disruption of ACE2 by the binding of severe acute respiratory syndrome coronavirus 2 (SARS-CoV-2). According to their article, SARS-CoV-2 binds and degrades ACE2, reducing the ability to regulate the renin-angiotensin system (RAS), eventually increasing its activity, which enhances the distal delivery of sodium and water to collecting tubules of the kidney and the excretion of potassium.

Coronavirus activates severe protein catabolism, resulting in wasting and loss of skeletal muscle mass, and leads to decreased total protein levels. This was corroborated by Ali and Kunugi, who suggested that degradation of body proteins is due to a complex interaction between inflammatory cytokines, reactive oxygen species, and liver damage secondary to hypercytokinemia, which ultimately results in hypoproteinemia. In some patients with symptoms of anorexia/vomiting and diarrhea, inadequate food intake also leads to hypoproteinemia [12]. Inflammation causes the escape of serum albumin into the interstitial space due to increased capillary permeability and eventually leads to an increased volume distribution of albumin. Thus, hypoalbuminemia is also precipitated by COVID-19 infection. The primary objective of our study was to correlate the investigations in both groups (COVID-19 patients with and without comorbid conditions with CT chest grades and within groups).

## Materials and methods

Study design

This was a single-center, retrospective, cross-sectional study conducted at the Institute of Biochemistry, Madras Medical College, by reviewing the electronic records of 1,000 reverse transcription-polymerase chain reaction (RT-PCR)-confirmed COVID-19-positive patients admitted to our hospital. The duration of the study was from May 10 to October 10, 2020. Out of 1,000 patients, 500 COVID-19 patients were selected with comorbid conditions such as T2DM, systemic hypertension (SHT), coronary artery disease (CAD), chronic obstructive pulmonary disease (COPD), and cerebrovascular accident (CVA) (Group I), and 500 COVID-19-positive patients were selected with no comorbid conditions (Group II). The study was approved by the institutional ethics committee.

Data collection

We collected data based on age, sex, and laboratory investigations, such as neutrophil-lymphocyte ratio (NLR), serum sodium, potassium, C-reactive protein (CRP), ferritin, urea, creatinine, total protein, and albumin, on the day of admission before treatment. CT chest findings were stratified into four grades based on the severity score used by our radiology institute. The four degrees of severity were obtained by categorizing each CT finding as follows: grade 0 (absent; total score 0); grade 1 (mild; total score 1); grade 2 (moderate; total score 2); and grade 3 (severe; total score ≥3) [13,14].

Statistical analysis

Statistical data were analyzed using the Statistical Package for the Social Sciences (SPSS) version 16 (SPSS Inc., Chicago, Illinois).

## Results

**Figure 1:**
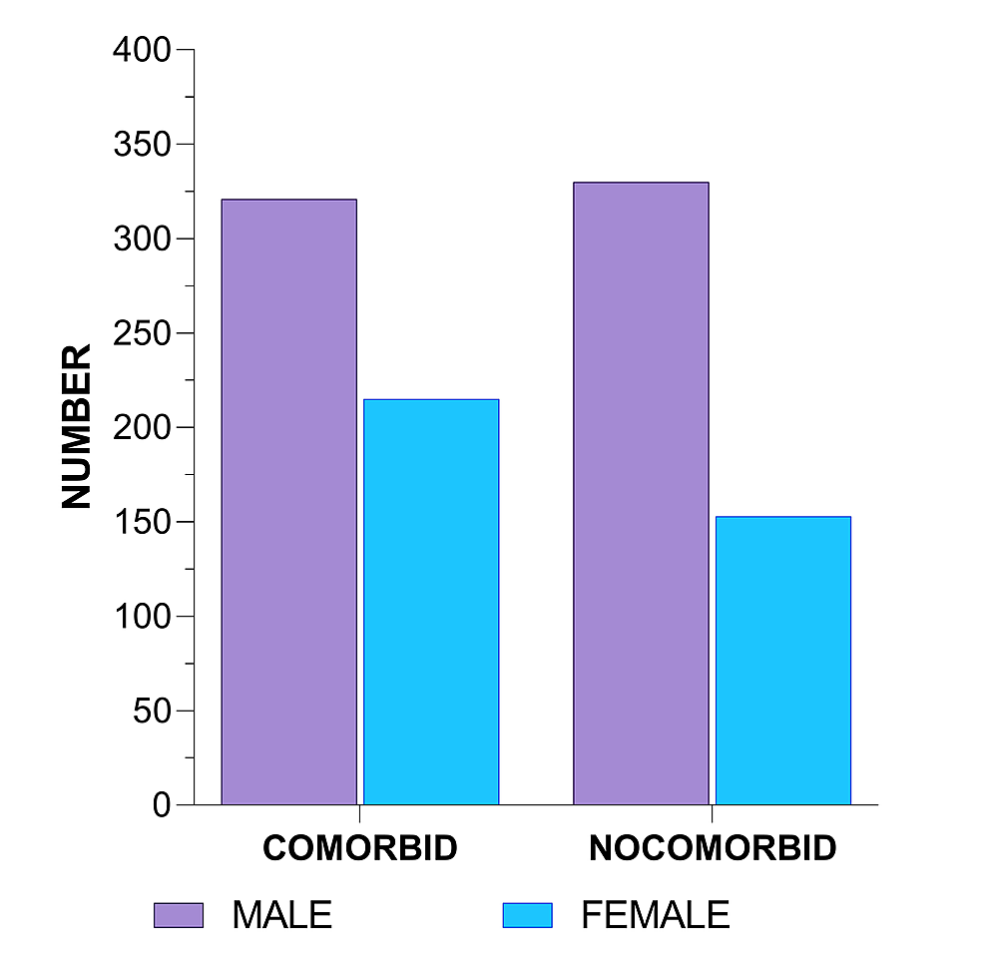
Sex distribution of the study subjects.

**Figure 2:**
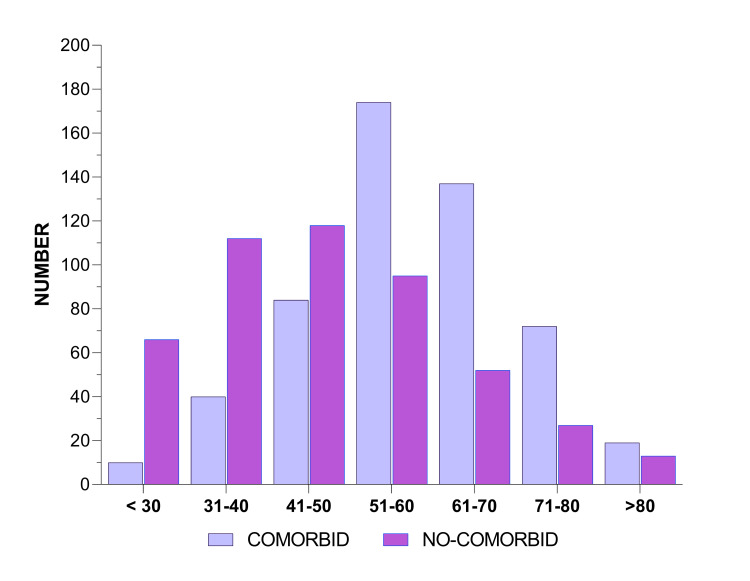
Age distribution of the study subjects.

In our study, male patients were more affected than female patients within all the grades of lung CT (Figures 1, 2). Among COVID-19 patients with comorbid conditions, there was no significant correlation with the NLR and urea, but significant p-values of 0.019, 0.027, 0.00, 0.00, 0.043, 0.001, and 0.000 were obtained for sodium, potassium, CRP, ferritin, creatinine, total protein, and albumin, respectively, among which CRP and ferritin showed the highest significance (Table 1). Patients with no comorbid conditions showed grade I CT changes mainly observed in the age group of up to 30 and 31-40 years (Table 2). Grade II, III, and IV CT changes were more common in the 41-50 and 51-60 age groups, even without comorbid conditions. When comparing the comorbid conditions with various grades of CT changes, serum sodium (hyponatremia) showed a significance of p = 0.011 in grade I and grade II, while serum potassium (hypokalemia) showed a significance of p = 0.019 in grade III and grade IV during the early stages of COVID-19. CRP and ferritin had significant levels of 0.044, 0.44, and 0.003 and 0.000, 0.001, 0.000, 0.001, and 0.205 in grades I and II, respectively, which implies that the correlation of inflammatory markers such as CRP and ferritin is highly significant at all stages. In patients without comorbid conditions, there was no significant association with NLR, urea, or creatinine. However, sodium, potassium, CRP, ferritin total protein, and albumin showed significant p-values of 0.000, 0.009, 0.000, 0.000, 0.034, and 0.000, respectively.

**Table 1:** Comparison between CT chest grades 1-4 in the comorbid group.

	CT grade					
	Grade 1		Grade 2	Grade 3	Grade 4	
	Mean ± SD		Mean ± SD	Mean ± SD	Mean ± SD	Sig.
Neutrophil-lymphocyte ratio	7.9 ± 16.5		9.8 ± 17.2	11.1 ± 17.2	11.2 ± 12.8	0.31
Sodium	136.3 ± 5.9	134.1 ± 5.3		135.5 ± 5.3	135.6 ± 7.4	0.019
Potassium	4.4 ± 0.6	4.3 ± 0.6		4.5 ± 0.7	4.4 ± 0.8	0.027
C-reactive protein	54.6 ± 75.1	86.3 ± 88.6		96.5 ± 115.5	132.3 ± 137.0	0
Ferritin	303.5 ± 339.2	542.6 ± 456.9		650.0 ± 543.1	788.0 ± 812.3	0
Urea	36.5 ± 32.5	38.2 ± 28.5		44.1 ± 26.5	42.1 ± 29.0	0.105
Creatinine	1.0 ± 0.7	1.0 ± 0.8		1.3 ± 1.9	0.9 ± 0.5	0.043
Total protein	6.9 ± 0.6	6.7 ± 0.6		6.6 ± 0.6	6.6 ± 0.7	0.001
Albumin	3.8 ± 0.5	3.6 ± 0.5		3.5 ± 0.4	3.5 ± 0.6	0

**Table 2:** Comparison between CT chest grades 1-4 in the non-comorbid group.

	Grade 1		Grade 2	Grade 3	Grade 4	
	Mean ± SD		Mean ± SD	Mean ± SD	Mean ± SD	Sig.
Neutrophil-lymphocyte ratio	4.8 ± 8.5	7.3 ± 12.0		6.4 ± 4.6	7.2 ± 9.9	0.093
Sodium	138.5 ± 4.8	136.9 ± 4.1		136.1 ± 4.6	137.0 ± 4.7	0
Potassium	4.2 ± 0.7	4.3 ± 0.6		4.3 ± 0.7	4.5 ± 0.6	0.009
C-reactive protein	34.6 ± 58.0	66.2 ± 97.5		96.6 ± 100.1	103.1 ± 79.6	0
Ferritin	303 ± 367.8	575.3 ± 610.9		677.0 ± 531.2	712.1 ± 486.6	0
Urea	32.6 ± 17.5	30.8 ± 13.4		35.1 ± 24.6	40.0 ± 23.9	0.013
Creatinine	0.9 ± 0.6	0.9 ± 0.8		1.1 ± 1.7	0.9 ± 0.4	0.225
Total protein	6.9 ± 0.6	6.8 ± 0.6		6.7 ± 0.6	6.7 ± 0.7	0.034
Albumin	3.8 ± 0.5	3.8 ± 0.5		3.6 ± 0.4	3.5 ± 0.5	0

## Discussion

Male patients were more affected than female patients within all the grades of lung CT. Bwire [15] suggested that testosterone increases the ACE2 receptor in the body. However, in females, soluble ACE2 receptors are induced by estrogen, which permits higher anti-inflammatory capacities, resulting in females having a reduced risk and severity of coronavirus infection than men. The comorbid conditions were found at 50-60 years of age. This was supported by Yanez et al. [16] in their study, which demonstrated that there was a striking increase in COVID-19 mortality rates in the elderly in proportion to age when compared to the rates of younger individuals. The logical explanation was that the number of comorbidities, such as hypertension, diabetes, obesity, cardiovascular disease, and changes associated with immunosenescence, which increase with age, enhanced vulnerability to infection, resulting in the disproportionately higher mortality due to COVID-19 in older patients [16].

Grade I CT changes were mainly observed in the age group up to 30 and 31-40 years. Grade II-IV CT changes were more frequent in the 41-50 and 51-60 age groups, even in the absence of comorbid conditions. This was supported by Farghaly and Makboul [17], who demonstrated that in patients less than 30 years of age, the correlation between age and CT score in both sexes was highly statistically significant at the lowest CT score; however, the highest CT severity score was detected in patients above 50 years of age. Thus, age can be considered a significant risk factor for the severity of COVID-19 in both sexes. Age-related CT changes were best explained by Wang et al., who found that CT images of elderly patients with basic diseases (hypertension, diabetes, hepatitis B, and COPD) were characterized by multi-affected lobes, subpleural lesions, crazy paving patterns, bronchodilatation, and pleural thickening when compared to those of young patients, particularly in severe/critical types [4].

In COVID-19 patients with comorbid conditions, statistically significant changes were obtained for sodium, potassium, CRP, ferritin, creatinine, total protein, and albumin. This observation was supported by the outcome of the meta-analysis, which showed that elevated systemic inflammation associated with liver damage is higher in patients with severe forms of COVID-19 infection [18]. In the presence of the most common comorbid conditions, such as HT, T2DM, obesity, and COPD, patients affected by COVID-19 had increased respiratory problems, poorer short-term outcomes, and a higher risk of death. T2DM patients with poor blood glucose control and HT or pre-existing CVD had substantially increased risks of morbidity and mortality.

CRP and ferritin had significance levels, which imply that the correlation of inflammatory markers such as CRP and ferritin is highly significant at all stages. Saeed. et al. [19] stated that the serum CRP level had a significant correlation with CT severity and could also be considered a predictive marker for the probability of disease progression even at an early stage of treatment. The authors also mentioned that serum ferritin is a vital mediator of immune dysregulation, and its level was closely linked to the severity of the disease.

Although creatinine showed overall significance, it did not project individual and CT-related changes. Even though there was no relationship between creatinine and CT changes, the risk of AKI associated with COVID-19 patients was as low as 7%. It was also mentioned that the etiology of AKI in the setting of COVID-19 is multifactorial, with prerenal factors such as volume depletion due to fever and poor oral intake, as well as hyperinflammatory syndrome leading to cytokine-mediated renal tubular injury in relation to various CT grades [20].

Serum albumin levels were predictive of adverse outcomes in patients with confirmed COVID-19. In our study, the majority showed hypoalbuminemia and hypoproteinemia, and the total protein and albumin showed a significance of 0.003 and 0.002 in stage III, and 0.003 and 0.001, respectively, in stage IV of CT grades, which implies the involvement of the liver only in the later stages of the disease, as it is an organ that controls all metabolic pathways, especially for proteins. This was supported by Kheir et al., [21] who found that albumin has significant antioxidant properties and plays a critical role during sepsis since oxygen free radicals can result in tissue ischemia, reperfusion injury, and an intense systemic inflammatory response. This implies that patients with hypoalbuminemia have diminished immunological responses during sepsis as well as in sepsis-associated complications such as venous thromboembolic events (VTE) and acute respiratory distress syndrome (ARDS) and, therefore, experience increased mortality. Moreover, albumin exhibits anticoagulant effects due to its capability to bind to antithrombin, which has an inhibitory effect on platelet aggregation. Therefore, in addition to prior known biomarkers (CRP, NLR, ferritin, and other biomarkers), serum albumin levels might help in prognostic risk stratification.

The interaction between inflammatory markers and insulin insensitivity enhances body protein degradation, resulting in decreased body protein levels. Among COVID-19 patients without comorbid conditions, sodium, potassium, CRP, ferritin, total protein, and albumin showed significant changes. This observation is in accordance with Doğan et al. [22], who observed that low albumin levels also decreased the levels of Na and K in COVID-19 patients. Although the gold standard test for the identification of COVID-19 patients is RT-PCR using throat and nasal swabs, in addition to CT chest, serological tests for electrolytes, inflammatory markers, total protein, and albumin also proved their value in the diagnosis and prognosis of COVID-19 patients, even with mild symptoms. Recovered COVID-19 patients who have features of AKI or acute renal failure (ARF) should be monitored regularly since they are at increased risk of developing chronic end-stage kidney disease.

Limitations

The data analyzed in this study were obtained from a single center and the data used are secondary that are available from the case records. If urinary investigations and serial measurements of creatinine could have been traced, more early involvement of renal pathology could have been identified. Inclusion of parameters such as D-dimer, ECG, echocardiogram (ECHO), and procalcitonin could have identified patients who might progress to myocarditis and sepsis, as these are also precipitating factors for AKI. However, these parameters were not available consistently in the case records of the selected patients.

## Conclusions

In conclusion, our study demonstrated that COVID-19 can cause mild to moderate renal impairment in both comorbid and non-comorbid groups. It was shown in our study that previous kidney disease could represent a risk factor for SARS-CoV-2, which also infects the normal kidneys and may cause AKI. Nevertheless, one of the most common organ dysfunctions in COVID-19 is AKI, and a large-scale prospective randomized study is needed to fully understand this issue.
